# Prognostic Impact of Metabolic Syndrome in Patients With Heart Failure: A Meta-Analysis of Observational Studies

**DOI:** 10.3389/fcvm.2021.704446

**Published:** 2021-06-24

**Authors:** Zhuo-Ming Huang, Wen-Rong Chen, Qi-Wen Su, Zhuo-Wen Huang

**Affiliations:** Department of Internal Medicine, Xingtan Hospital Affiliated to Shunde Hospital of Southern Medical University, Foshan, China

**Keywords:** heart failure, metabolic syndrome, all-cause mortality, cardiovasclar disease, prognosis

## Abstract

**Background:** The metabolic syndrome (MS) is significantly associated with the risk of incident heart failure (HF). However, there are still great controversies about the impact of MS on the prognosis in patients with established HF. This meta-analysis aimed to ascertain the effect of MS on the prognosis in patients with HF.

**Methods:** We searched multiple electronic databases, including PubMed, Opengrey, EMBASE, and Cochran Library, for potential studies up to February 15, 2021. Observational studies that reported the impact of MS on the prognosis in patients with established HF were included for meta-analysis.

**Results:** Ten studies comprising 18,590 patients with HF were included for meta-analysis. The median follow-up duration of the included studies was 2.4 years. Compared with HF patients without MS, the risk of all-cause mortality and cardiovascular mortality was not increased in HF with MS (HR = 1.04, 95% CI = 0.88–1.23 for all-cause mortality; HR = 1.66, 95% CI = 0.56–4.88 for cardiovascular mortality, respectively). However, there was a significant increase in composited cardiovascular events in the HF patients with MS compared with those without MS (HR = 1.73, 95% CI = 1.23–2.45).

**Conclusions:** In patients with established HF, the presence of MS did not show an association on the risk of all-cause mortality or cardiovascular mortality, while it may increase the risk of composite cardiovascular events.

## Introduction

Metabolic syndrome (MS) is a cluster of cardiovascular risk factors characterized by insulin resistance, central obesity, elevated blood pressure, and dyslipidemia (1, 2). Epidemiological studies have shown that MS and its components are highly prevalent and significantly associated with the development of diabetes and cardiovascular disease (CVD) (2). Heart failure (HF) is a growing global public health burden, attributed to significantly increased mortality worldwide (3). Detection for the pathophysiological characteristics and novel treatment targets would be important for management of HF (4–6). It is well-documented that MS is an independent risk factor in the development of HF (7). Individuals with MS were associated with a 2-fold risk of HF incidence compared with those without MS (8). However, there are still great controversies about the impact of MS on the prognosis in patients with established HF. Some studies had reported that HF patients with MS had a significantly reduced risk of in-hospital mortality (9), as well as long-term all-cause mortality (10, 11), compared with HF patients without MS. However, such association was not documented in other studies (12, 13), raising concern of whether such “epidemiologic paradox” was true in the HF–MS relationship.

Given these inconsistent results and controversies, we performed a meta-analysis to ascertain the impact of MS on the prognosis in patients with HF, focusing on all-cause mortality, cardiovascular mortality, and composite cardiovascular events.

## Methods

### Search Strategy and Selection Criteria

This study was performed under the recommendations of the Meta-analysis of Observational Studies in Epidemiology group (14). We searched multiple electronic databases, including PubMed, Medline, EMBASE, and Cochran Library, for potential related studies up to February 15, 2021. Items including “metabolic syndrome,” “syndrome X,” “insulin resistance syndrome,” “heart failure,” “myocardial failure,” “cardiac failure,” “cardiac dysfunction,” or “myocardial dysfunction” were searched using a combined MeSH terms and text word search strategy. No language restriction was set in the search strategy. However, we restricted the search to human studies by using the “Humans” filters. The reference lists of included studies were reviewed to identify other potential associated research.

Inclusion criteria of studies for meta-analysis were the following: (1) observational studies (cohort studies, nest case–control studies, or *post-hoc* analysis of randomized controlled trials) with a follow-up duration ≥6 months; (2) the prognoses in HF patients were reported in those with MS, compared with those without MS. Exclusion criteria were the following: (1) cross-sectional studies without follow-up evaluation; (2) duplicated publications derived from the same observational study. When duplicated publications were derived from the same observational study, we only included the latest published article for analysis.

### Data Extraction and Quality Assessment

Two researchers (ZH and WC) screened the titles and abstracts of all the retrieved items and reviewed the full manuscripts of potentially relevant studies independently. Key information of the included studies, such as authors, study design, race, definition and prevalence of MS, sample size, sex proportion, age, follow-up duration, adjusted confounders, and outcome events were recorded. We also contacted the corresponding authors of the original studies for any additional data if required.

We evaluated the quality of the included studies according to the Newcastle–Ottawa Quality Assessment Scale (NOS) for cohort studies (15). The NOS judged the quality based on selection, comparability, and exposure/outcome, with the highest score up to 9. In the present analysis, included studies were graded in quality as good, fair, or poor if they were awarded ≥7, 4–6, or <4 scores, respectively (16, 17).

### Data Synthesis and Analysis

The primary outcome in our study was the risk of all-cause mortality in HF patients with MS compared with those without MS. The secondary outcomes included the risk of cardiovascular mortality and composite cardiovascular events, respectively. Hazard ratios (HRs) and 95% confidence intervals (CIs) adjusted for the maximal number of confounders were extracted for analysis. If relative risks (RRs) were reported, they were considered as approximate estimate for HRs and used for meta-analysis (18). In situations where outcomes were presented as odds ratios (ORs), data were converted to RRs for analysis, according to previously published method (19). *I*^2^ statistics were used to test heterogeneity among studies, and an *I*^2^ > 50% was considered to be with significant heterogeneity. We used the inverse variance approach to combine the log HRs and corresponding standard errors (SE). A random-effects model was used for meta-analysis if there was significant heterogeneity. Otherwise, a fixed-effects model was used. Sensitivity analyses were performed by omitting one study and recalculating the estimates of HRs at a time or interchange using random-effects models and fixed-effects models for the meta-analysis. Publication bias was evaluated by inspecting funnel plots for the primary outcome in which the lnHR was plotted against SE and also tested by using Egger's and Begg's tests. Subgroup analyses were performed according to participant's age, follow-up duration, race, the severity of HF, adjusted confounders, and definition of MS.

Analyses were performed using RevMan 5.3 (The Cochrane Collaboration, Copenhagen, Denmark) and Stata 12.0 (StataCorp LP, College Station, TX, USA) for Egger's and Begg's tests. A *p* < 0.05 was set for statistical significance.

## Results

### Studies Retrieved and Characteristics

**Figure 1:**
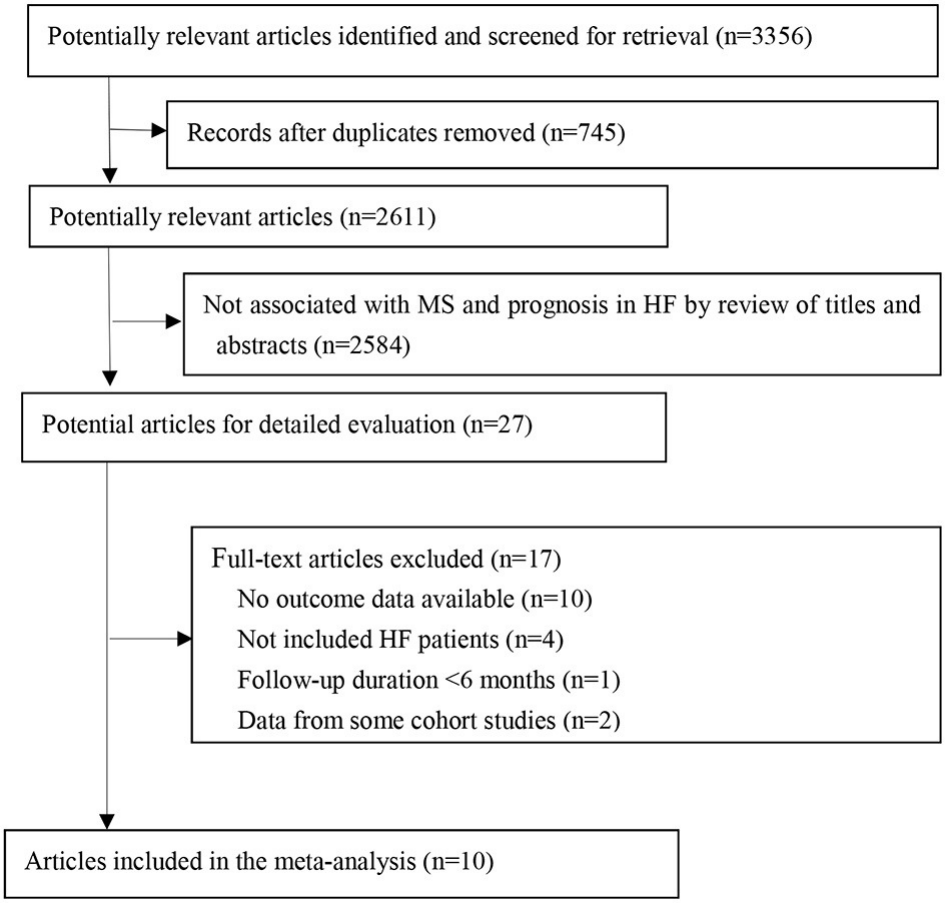
Flow diagram of study selection. HF, heart failure; MS, metabolic syndrome.

A total of 3,356 items were detected from the online electronic databases. After screening the titles and abstracts, we found that 27 articles were qualified for a full review. Finally, we included 10 studies comprising 18,590 patients with HF for meta-analysis (Figure 1) (10–13, 20–25). The median follow-up duration of the included studies was 2.4 years.

In the 10 studies, 8 were from the United States and Europe; 2 were from Asia. The key messages of the included studies are presented in Table 1. All studies included advanced HF patients, except one study that included patients with asymptomatic HF (stage A/B HF). Five, four, and one study defined MS according to the Third Adult Treatment Panel Report of the National Cholesterol Education Program (NCEP-ATP III), the International Diabetes Federation guideline (IDF), and the Japanese Committee for the Diagnostic Criteria of MS, respectively. Two studies were published as meeting abstracts, and full articles were not available. Therefore, quality assessment was only performed in the eight studies with full published articles. According to NOS criteria, six studies were graded as good quality and two as fair quality (Supplementary Table 1).

**Table 1:** Characteristics of the included studies.

**References**	**Design**	**Region**	**Patients characteristics**	**Sample (female %)**	**Age (years) (mean)**	**Follow-up duration (years)**	**Definition and prevalence of MS**	**Events for analysis**	**Risk factors adjusted**
Hassan et al. (10)	Retrospective cohort study	US	Hospitalized HF	886 (20.1)	64.9	2.4	NCEP-ATP III (68.3%)	All-cause mortality	Age, sex, race, LVEF, hemoglobin, eGFR, TC, LDL-C, AF, QRS duration on electrocardiogram, health care site, ACEI/ARB
Tamariz et al. (12)	Prospective cohort study	US	HFrEF	865 (37.0)	55.1	2.6	NCEP-ATP III (40%)	All-cause mortality	Demographics, use of ACEI, *β*-blocker, hematocrit, creatinine, educational level, and LVEF.
Ahmed et al. (20)^#^	Retrospective cohort study	US	HF received ICD	171 (NA)	71.0	2.3	NCEP-ATP III (25.1%)	CVD mortality	Age, sex
Bajraktari et al. (21)^#^	Prospective cohort study	Sweden	Congestive HF	188 (NA)	62.0	1.5	NCEP-ATP III (44.1%)	Composite CVD	Multivariate adjusted, but confounder were not reported
Perrone-Filardi et al. (11)	*post-hoc* analysis of RCT	Italy	Symptomatic HF (NYHA II–IV)	6,648 (21.8)	67.2	3.9	IDF (18.2%)	All-cause mortality CVD mortality	Age, HR, LVEF, previous MI, CABG, PAD, percentage of patients with pacemaker, ICD, COPD, ischemic HF, ARB, diuretics, and CCB.
Carrubba et al. (13)	Prospective cohort study	Italy	Stage A/B HF (asymptomatic HF)	1,920 (43.8)	60.0	0.9	IDF (13.4%)	All-cause mortality Composite CVD CVD mortality	Left ventricular dysfunction, age, gender, history of MI.
Tadaki et al. (22)	Prospective cohort study	Japan	Stage C/D CHF.	4,762 (32.0)	68.8	3.2	Japanese criteria (41.3%)	All-cause mortality Composite CVD	Age, sex, BMI, BNP, LVEF, SBP, DBP and HR.
Vest et al. (23)	Retrospective cohort study	US	HFrEF	1,953 (26.0)	55	5.1	NCEP-ATP III (37%)	All-cause mortality	Age, sex, LVAD, transplantation, and factors unbalanced between the +MS and –MS subgroups
Welnicki et al. (24)	Retrospective cohort study	Poland	Stage C/D CHF without AF	893 (31.0)	63.6	1.0	IDF (13.0%)	All-cause mortality	Unadjusted
Cetin et al. (25)	Retrospective cohort study	Turkey	HFrEF	304 (17.8)	47.4	1.3	IDF (36.2%)	All-cause mortality	Unadjusted

*ACEI, angiotensin-converting enzyme inhibitor; AF, atrial fibrillation; ARB, angiotensin receptor blockers, BMI, body mass index; BNP, B-type natriuretic peptide; CCB, calcium channel blockers; COPD, chronic obstructive pulmonary disease; CVD, cardiovascular disease; DBP, diastolic blood pressure; eGFR, estimated glomerular filtration rate; HF, heart failure; HFrEF, HF with reduced ejection fraction; HR, heart rate; ICD, Implantable cardioverter-defibrillator; IDF, International Diabetes Federation; LDL-C, low-density lipoprotein cholesterol; LVEF, left ventricular ejection fraction; MI, myocardial infarction; MS, Metabolic Syndrome; NA, not available; NCEP-ATP III: the Third Adult Treatment Panel Report of the National Cholesterol Education Program; PAD, peripheral artery disease; RCT, randomized controlled trial; SBP, systolic blood pressure; TC, total cholesterol*.

# *Meeting abstract*.

### Association Between MS and Prognosis in Patients With HF

**Figure 2:**
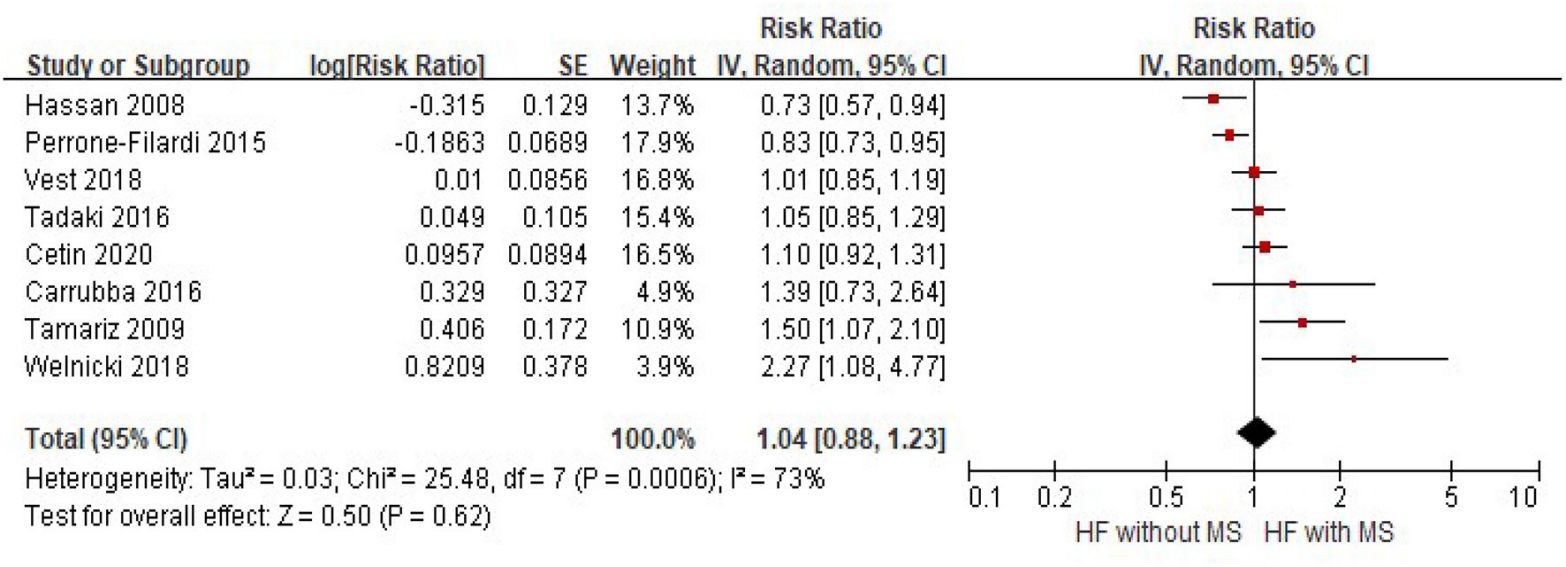
The association of MS and the risk of all-cause mortality in HF patients. CI, confidence interval; HF, heart failure; MS, metabolic syndrome.

There were eight studies that provided data for analysis of the association between MS and all-cause mortality in patients with HF. Due to the significant heterogeneity among the included studies (*I*^2^ = 73%, *p* < 0.001), a random-effects model was used to combined the pooled estimates. Compared with patients without MS, the risk of all-cause mortality was not increased in HF patients with MS (HR = 1.04, 95% CI = 0.88–1.23, *p* = 0.62) (Figure 2). No evidence of publication bias was found by visual inspection of the funnel plot (Supplementary Figure 1) or using Egger's test or Begg's (all *p* > 0.05).

**Figure 3:**
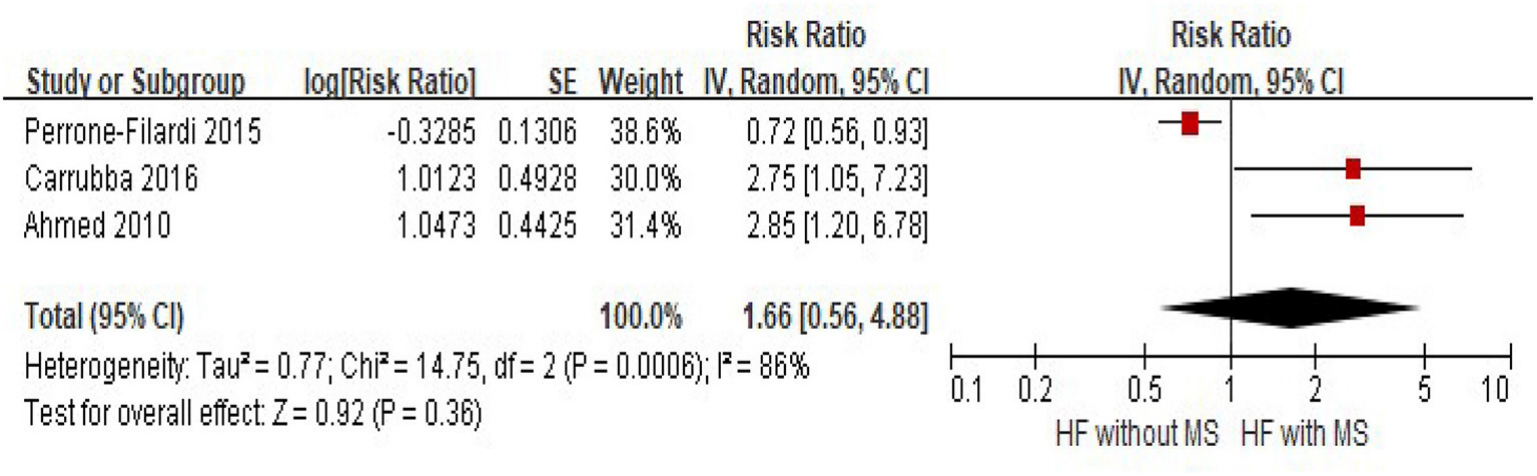
The association of MS and the risk of cardiovascular mortality in HF patients. CI, confidence interval; HF, heart failure; MS, metabolic syndrome.

Three studies provided the risk of cardiovascular mortality in HF patients with and without MS, and significant heterogeneity was observed among the studies (*I*^2^ = 86%, *p* < 0.001). Random-effects models meta-analysis showed that there was no significant difference in cardiovascular mortality between the groups (HF with MS vs. HF without HF, HR = 1.66, 95% CI = 0.56–4.88, *p* = 0.36) (Figure 3).

**Figure 4:**
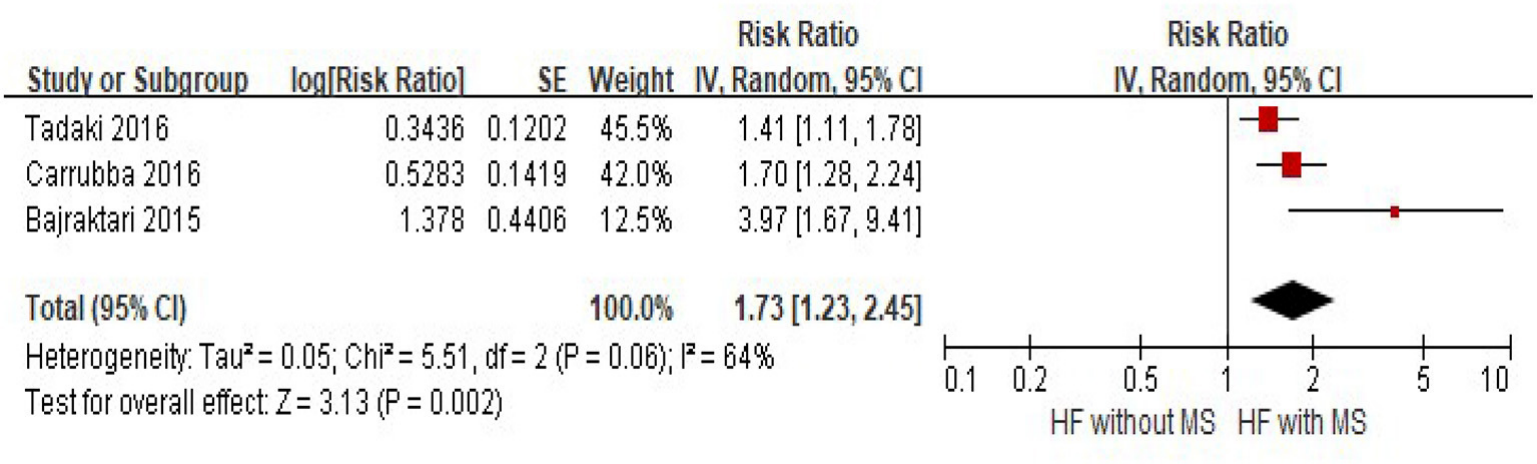
The association of MS and the risk of composite cardiovascular events in HF patients. CVD, cardiovascular disease; CI, confidence interval; HF, heart failure; MS, Metabolic Syndrome.

Although significant heterogeneity existed among three studies that reported composited cardiovascular events (*I*^2^ = 64%, *p* = 0.06), pooled estimates showed that there was a substantial increase in composited cardiovascular events in HF patients with MS, compared with those without HF (HR = 1.73, 95% CI = 1.23–2.45, *p* = 0.002) (Figure 4).

### Subgroup Analyses and Sensitivity Analyses

The subgroup analyses for the relative risk of all-cause mortality in HF patients with MS are presented in Table 2. Patients with HF and MS did not show an association with all-cause mortality in all the subgroup analyses. Furthermore, there was no significant heterogeneity observed among subgroup comparisons (all *p* > 0.15).

**Table 2:** Subgroup analyses of the association between MS and risk of all-cause mortality in HF patients.

	**Number of studies**	**HF** **+** **MS vs. HF** **+** **non-MS**	
		**HR (95% CI)**^**#**^	*p-*value^*^
Participants' age			0.20
≤60 years	4	1.14 (0.97, 1.33)	
>60 years	4	0.94 (0.73, 1.20)	
Follow-up duration			0.15
≤3 years	6	1.14 (0.90, 1.43)	
>3 years	2	0.91 (0.75, 1.10)	
Race			0.86
Asian	2	1.08 (0.94, 1.23)	
Non-Asian	6	1.05 (0.83, 1.33)	
Severity of HF			0.37
Asymptomatic HF	1	1.39 (0.73, 2.64)	
Symptomatic HF	7	1.03 (0.87, 1.22)	
Adjusted confounders			0.30
Unadjusted	2	1.44 (0.72, 2.86)	
Multivariable adjusted	6	0.99 (0.83, 1.18)	
Definition of MS			0.70
NCEP-ATP III	3	1.11 (0.82, 1.50)	
IDF	4	1.02 (0.72, 1.43)	

* *For heterogeneity among subgroups*.

*HF, heart failure; HR, hazard ratio; IDF, International Diabetes Federation; MS, Metabolic Syndrome; NCEP-ATP III, the Third Adult Treatment Panel Report of the National Cholesterol Education Program*.

Several sensitivity analyses confirmed that there was no significant association between MS status and risk of all-cause mortality, using fixed-effects models instead of random-effects models or recalculating the estimated risk by omitting one study at a time.

## Discussion

To the best of our knowledge, this is the first meta-analysis exploring the impact of MS on the prognosis in patients with established HF. Our data showed that MS is not associated with the risk of all-cause mortality or cardiovascular mortality in HF patients, while an increased risk of composite cardiovascular events was observed. Therefore, this study challenges the “epidemiologic paradox” in the relationship between HF and MS.

### Clinical Implications

Observational studies had reported that in patients with established HF, risk factors that contribute to the development of CVD and HF, including hypertension, obesity, and hypercholesterolemia, might have a protective effect on prognosis. This phenomenon was termed as “reverse epidemiology” or “risk factor paradox” (26). However, the nature of observational studies could not exclude “reverse causation” in such observed associations; i.e., it may not be that obesity, hypertension, and hypercholesterolemia can play a protective role, while low body mass index (BMI), blood pressure, or serum cholesterol are “markers” of a severe pathological condition in HF, closely linked to cachexia, frailty, and death (7). In our study, the presence of MS was not associated with a favorable prognosis in the HF population, and no evidence of a “metabolic syndrome survival paradox” was found. Our results were supported by the study of Ozcan et al. (25). which showed that the risk of all-cause mortality was not statistically different in HF patients with and without MS. However, in HF patients with “reverse metabolic syndrome” (i.e., low BMI, blood pressure, and total cholesterol than normal level), the risk of all-cause mortality was increased in metabolically healthy patients. In this view, we should not recommend that MS is a protective factor; rather we should advocate that in HF patients with “reverse metabolic syndrome,” targeting catabolic syndrome by nutritional support and anti-inflammatory treatment should be considered in future studies.

Actually, we found that in HF with MS, the risk of composite cardiovascular events was increased compared with those without MS. This finding further supports that MS is an independent risk of atherosclerotic cardiovascular events (2), even in patients with established HF. Besides the effect of the cluster of atherosclerotic risk factors, activation of the sympathetic nervous system and renin–angiotensin system may also be involved in this process. It had been proposed that in HF patients with MS, activation of the sympathetic nervous system and renin–angiotensin system was higher than those without MS (27, 28). Our study also found that the prevalence of MS was up to 13.0–68.3%, based on different definitions. Considering the high prevalence and association with increased risk of composite cardiovascular events, proper management of this population would be of important clinical effect. During the past decade, the treatment of HF had made great progress, and many patients with severe HF can survive for a long time. In this situation, prevention of CVD in established HF with MS should be considered.

### Limitations

Several limitations of the current study should be mentioned. First, the definitions of MS were different in the included studies. However, our subgroup analysis showed that the risk of all-cause mortality was not increased in HF with MS; neither was defined according to the IDF nor NCEP-ATP III criteria. Second, the definitions of HF among the included studies were with significant heterogeneity. The etiologies of HF are also unknown in the studies. However, we did not observe a difference in the subgroup analysis according to the severity of HF. Even in patients with advanced HF, the risk of all-cause mortality was not associated with the presence of MS. Third, MS is a cluster of cardiovascular risk factors, including obesity, elevated blood pressure, elevated blood glucose, and dyslipidemia. Due to limited data, we did not evaluate the effect of the individual components of MS on the prognosis in HF. Fourth, although we reported that the risk of composite cardiovascular events was increased in HF with MS, only three studies were available for the meta-analysis of this outcome. Further studies are needed to explore the association between MS and the risk of CVD in patients with established HF.

## Conclusions

In patients with established HF, the presence of MS did not show a protective effect on the risk of all-cause mortality or cardiovascular mortality, while it may increase the risk of composite cardiovascular events.

## Data Availability Statement

The raw data supporting the conclusions of this article will be made available by the authors, without undue reservation.

## Author Contributions

Z-MH and W-RC: research idea, study design, and data acquisition. Q-WS and Z-WH: data analysis/interpretation and statistical analysis. W-RC: supervision and mentorship. All authors contributed important intellectual content during manuscript drafting or revision and accept accountability for the overall work by ensuring that questions pertaining to the accuracy or integrity of any portion of the work are appropriately investigated and resolved.

## Conflict of Interest

The authors declare that the research was conducted in the absence of any commercial or financial relationships that could be construed as a potential conflict of interest.

## Abbreviations

CIs: confidence intervals
CVD: cardiovascular disease
HF: heart failure
HRs: hazard ratios
IDF: International Diabetes Federation
MS: metabolic syndrome
NCEP-ATP III: Third Adult Treatment Panel Report of the National Cholesterol Education Program
NOS: Newcastle–Ottawa Quality Assessment Scale
RRs: relative risks
SE: standard errors.

## References

[1] Beltran-Sanchez H Harhay MO Harhay MM McElligott S. Prevalence and trends of metabolic syndrome in the adult U.S. population, 1999-2010. J Am Coll Cardiol. (2013) 62:697–703. 10.1016/j.jacc.2013.05.064 23810877 PMC3756561

[2] Mottillo S Filion KB Genest J Joseph L Pilote L Poirier P. The metabolic syndrome and cardiovascular risk a systematic review and meta-analysis. J Am Coll Cardiol. (2010) 56:1113–32. 10.1016/j.jacc.2010.05.034 20863953

[3] Cai X Liu X Sun L He Y Zheng S Zhang Y. Prediabetes and the risk of heart failure: a meta-analysis. Diabetes Obes Metab. (2021) 10.1111/dom.14388. [Epub ahead of print]. 33769672

[4] Li W Huang A Zhu H Liu X Huang X Huang Y. Gut microbiota-derived trimethylamine N-oxide is associated with poor prognosis in patients with heart failure. Med J Aust. (2020) 213:374–9. 10.5694/mja2.50781 32959366

[5] Yang S Chen H Tan K Cai F Du Y Lv W. Secreted frizzled-related protein 2 and extracellular volume fraction in patients with heart failure. Oxid Med Cell Longev. (2020) 2020:2563508. 10.1155/2020/2563508 32454934 PMC7229555

[6] Wu J Zheng H Liu X Chen P Zhang Y Luo J. Prognostic value of secreted frizzled-related protein 5 in heart failure patients with and without type 2 diabetes mellitus. Circ Heart Fail. (2020) 13:e7054. 10.1161/CIRCHEARTFAILURE.120.007054 32842761

[7] Arcopinto M Schiavo A Salzano A Bossone E D'Assante R Marsico F. Metabolic syndrome in heart failure: friend or foe? Heart Fail Clin. (2019) 15:349–58. 10.1016/j.hfc.2019.02.004 31079693

[8] Horwich TB Fonarow GC. Glucose, obesity, metabolic syndrome, and diabetes relevance to incidence of heart failure. J Am Coll Cardiol. (2010) 55:283–93. 10.1016/j.jacc.2009.07.029 20117431 PMC2834416

[9] Yoon HJ Ahn Y Kim KH Park JC Choi DJ Han S. The prognostic implication of metabolic syndrome in patients with heart failure. Korean Circ J. (2013) 43:87–92. 10.4070/kcj.2013.43.2.87 23508725 PMC3596669

[10] Hassan SA Deswal A Bozkurt B Aguilar D Mann DL Pritchett AM. The metabolic syndrome and mortality in an ethnically diverse heart failure population. J Card Fail. (2008) 14:590–95. 10.1016/j.cardfail.2008.03.004 18722325

[11] Perrone-Filardi P Savarese G Scarano M Cavazzina R Trimarco B Minneci S. Prognostic impact of metabolic syndrome in patients with chronic heart failure: data from GISSI-HF trial. Int J Cardiol. (2015) 178:85–90. 10.1016/j.ijcard.2014.10.094 25464226

[12] Tamariz L Hassan B Palacio A Arcement L Horswell R Hebert K. Metabolic syndrome increases mortality in heart failure. Clin Cardiol. (2009) 32:327–31. 10.2337/diacare.24.4.683 19569069 PMC6653189

[13] La Carrubba S Antonini-Canterin F Fabiani I Colonna P Pugliese NR Caso P. Prevalence and prognostic impact of metabolic syndrome in asymptomatic (stage A and B heart failure) patients. Metab Syndr Relat Disord. (2016) 14:187–94. 10.1089/met.2015.0143 26866978

[14] Stroup DF Berlin JA Morton SC Olkin I Williamson GD Rennie D. Meta-analysis of observational studies in epidemiology: a proposal for reporting. Meta-analysis Of Observational Studies in Epidemiology (MOOSE) group. JAMA. (2000) 283:2008–12. 10.1001/jama.283.15.2008 10789670

[15] Wells GA Shea B O'Connell D Robertson J Peterson J Welch V. The Newcastle-Ottawa Scale (NOS) for Assessing the Quality of Nonrandomised Studies in Meta-Analyses. Available online at: http://www.ohri.ca/programs/clinical_epidemiology/oxford.asp (accessed January 1, 2008)

[16] Cai X Zhang Y Li M Wu JH Mai L Li J. Association between prediabetes and risk of all-cause mortality and cardiovascular disease: updated meta-analysis. BMJ. (2020) 370:m2297. 10.1136/bmj.m2297 32669282 PMC7362233

[17] Huang Y Huang W Mai W Cai X An D Liu Z. White-coat hypertension is a risk factor for cardiovascular diseases and total mortality. J Hypertens. (2017) 35:677–688. 10.1097/HJH.0000000000001226 28253216 PMC5338886

[18] Yang Y Li W Zhu H Pan XF Hu Y Arnott C. Prognosis of unrecognised myocardial infarction determined by electrocardiography or cardiac magnetic resonance imaging: systematic review and meta-analysis. BMJ. (2020) 369:m1184. 10.1136/bmj.m1184 32381490 PMC7203874

[19] Cai X Zheng S Liu Y Zhang Y Lu J Huang Y. Nonalcoholic fatty liver disease is associated with increased risk of atrial fibrillation. Liver Int. (2020) 40:1594–600. 10.1111/liv.14461 32279432

[20] Ahmed I Nelson WB Dahiya R House CM Zhu DW. Metabolic syndrome predicts poor outcome in patients with systolic heart failure who received implantable-cardioverter defibrillator for primary prevention of sudden cardiac death. Heart Rhythm. (2010) 7:S252–53. 10.1016/j.hrthm.2010.03.031

[21] Bajraktari G Berisha G Bytyci I Haliti E Ibrahimi P Ahmeti A. The presence of metabolic syndrome predicts long-term outcome in heart failure patients. Eur Heart J. (2015) 36:831. 10.2337/dc08-1394

[22] Tadaki S Sakata Y Miura Y Miyata S Asakura M Shimada K. Prognostic impacts of metabolic syndrome in patients with chronic heart failure-a multicenter prospective cohort study. Circ J. (2016) 80:677–88. 10.1253/circj.CJ-15-0942 26794282

[23] Vest AR Young JB Cho L. The metabolic syndrome, cardiovascular fitness and survival in patients with advanced systolic heart failure. Am J Cardiol. (2018) 122:1513–9. 10.1016/j.amjcard.2018.07.024 30172361

[24] Welnicki MT Sliz DI Szeligowska J Duda-Król WB Chomiuk T Dabrowska D. The influence of metabolic syndrome coexistence on the prognosis of patients with heart failure without atrial fibrillation. analysis of Polish data from the pilot survey for the ESC Heart Failure Registry. Kardiol Pol. (2018) 76:794–6. 10.5603/KP.2018.0077 29652423

[25] Ozcan CE Cetin MS Ozbay MB Yaman NM Könte HC Ekizler FA. The other side of the medallion in heart failure: reverse metabolic syndrome. Nutr Metab Cardiovasc Dis. (2020) 30:2041–50. 10.1016/j.numecd.2020.06.027 32830019

[26] Gargiulo P Marsico F Renga F Dell'Aversana S Esposito I Marciano C. The metabolic syndrome in heart failure: insights to specific mechanisms. Heart Fail Rev. (2020) 25:1–7. 10.1007/s10741-019-09838-6 31414215

[27] Sarzani R Salvi F Dessi-Fulgheri P Rappelli A. Renin-angiotensin system, natriuretic peptides, obesity, metabolic syndrome, and hypertension: an integrated view in humans. J Hypertens. (2008) 26:831–43. 10.1097/HJH.0b013e3282f624a0 18398321

[28] Quarti TF Dell'Oro R Biffi A Seravalle G Corrao G Mancia G. Sympathetic overdrive in the metabolic syndrome: meta-analysis of published studies. J Hypertens. (2020) 38:565–72. 10.1097/HJH.0000000000002288 32132429

