# Supplementary material for: Prognostic Impact of Metabolic Syndrome in Patients With Heart Failure: A Meta-Analysis of Observational Studies
Source: Front Cardiovasc Med. 2021 Jun 24;8:704446. doi: 10.3389/fcvm.2021.704446 (PMC8263914; doi:10.3389/fcvm.2021.704446)
Supplement: Supplementary file 1 [file Data_Sheet_1.doc]

**Supplemental table 1. Quality Assessment of the Included Studies by NOS**

| **Study** | **Selection**  **(stars awarded)** | **Comparability (stars awarded)** | **Outcome**  **(stars awarded)** | **Quality (total stars)^*^** |
| --- | --- | --- | --- | --- |
| Hassan 2008 | 3 | 2 | 2 | Good (7) |
| Tamariz 2009 | 3 | 1 | 3 | Good (7) |
| Ahmed 2010^#^ | - | - | - | - |
| Bajraktari 2015^#^ | - | - | - | - |
| Perrone-Filardi 2015 | 3 | 2 | 2 | Good (7) |
| Carrubba 2016 | 3 | 2 | 2 | Good (7) |
| Tadaki 2016 | 4 | 2 | 2 | Good (8) |
| Vest 2018 | 3 | 1 | 3 | Good (7) |
| Welnicki 2018 | 3 | 1 | 2 | Fair (6) |
| Cetin 2020 | 3 | 1 | 2 | Fair (6) |

NOS, Newcastle–Ottawa Quality Assessment Scale (NOS) for cohort studies


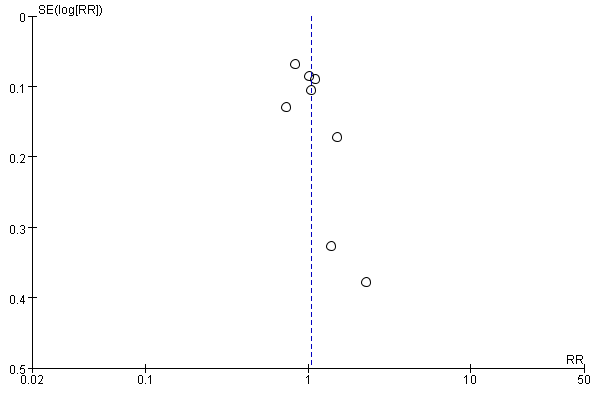


**Supplementary Figure 1. Funnel plot of comparison of risk of all-cause mortality in HF+MS compared with HF+non-MS group**

HF, heart failure; MS, metabolic syndrome
